# An ionic organic–inorganic hybrid: tetra­kis[bis­(1,10-phenanthroline)copper(I)] dodeca­tungstophosphate(V). Corrigendum

**DOI:** 10.1107/S1600536808005588

**Published:** 2008-03-05

**Authors:** Fan-Xia Meng, Hong-Bo Liu, Ya-Guang Chen

**Affiliations:** a Key Laboratory of Polyoxometallate Science of the Ministry of Education, College of Chemistry, Northeast Normal University, Changchun 130024, People’s Republic of China; b Department of Pharmaceutics, Changchun Medical College, Changchun 130031, People’s Republic of China

## Abstract

The chemical name in the title of the paper by Meng, Liu & Chen [*Acta Cryst.* (2008), E**64**, m106] is corrected.

## Abstract 2

Corrigendum to *Acta Cryst.* (2008), E**64**, m106.

In the paper by Meng, Liu & Chen [*Acta Cryst.* (2008), E**64**, m106], the chemical name in the title is incorrect. The correct chemical name should be ‘tetra­kis[bis­(1,10-phenanthroline)copper(I)] dodeca­tungstosilicate’.

